# Assessing genetic and agronomic gains in rice yield in sub-Saharan Africa: A meta-analysis

**DOI:** 10.1016/j.fcr.2022.108652

**Published:** 2022-10-15

**Authors:** Ali Ibrahim, Kazuki Saito

**Affiliations:** a Africa Rice Center (AfricaRice), Regional Station for the Sahel, B.P. 96, Saint-Louis, Senegal; b Africa Rice Center (AfricaRice), 01 B.P. 2551, Bouaké 01, Côte d′Ivoire

**Keywords:** Agronomy, Genetic improvement, Meta-analysis, *Oryza* spp

## Abstract

Research for development efforts for increasing rice yield in sub-Saharan Africa (SSA) have largely concentrated on genetic improvement and agronomy for more than 50 years. Here we perform the first meta-analysis to quantify genetic gain - yield increase through use of new variety and calculated by yield difference between new variety and variety popularly grown in the target site, and agronomic gain - difference in yield between improved agronomic practices and the control in SSA using 208 paired observations from 40 studies across 12 countries. Among the studies, 41 %, 34 %, and 25 % were from irrigated lowland, rainfed lowland, and rainfed upland rice, respectively. Seventy percent of the studies reported in this paper were conducted on research stations. In agronomic practices, inorganic fertilizer management practices accounted for 78 % of the studies, of which 48 % were nitrogen (N) management. In each study, we identified four types of varieties: check variety (VC), variety with highest yield in the control (VHC), variety with highest yield under improved agronomic practices (VHT), and variety with largest yield difference between improved agronomic practices and control (VHR). VHT was the same as VHC in 35 % of observations, whereas VHR and VHT were the same in 51 %. These indicate that it is possible to develop varieties adapted to different agronomic practices and high-yielding varieties tend to be responsive to improved agronomic practices. On average, total gain in yield with improved agronomic practices and VHT was 1.6 t/ha. Agronomic practice accounted for 75 % of the total variation in total yield gain with variety and agronomic practice by variety interaction responsible for 19 % and 6 %, respectively. Genetic gains in yield with VHC, VHT, and VHR were 0.7, 0.3, and −0.3 t/ha in control, and 0.4, 0.9, and 0.5 t/ha in improved agronomic practices. Agronomic gain in yield averaged 0.5, 0.8, 1.4, and 1.6 t/ha in VHC, VC, VHT, and VHR, respectively. Agronomic gain in yield of VHT was higher than genetic gain under improved agronomic practices in 54 % of observations. Agronomic gain was highest in irrigated lowland rice, followed by rainfed lowland rice. Higher agronomic gain in yield was also associated with larger difference in N application rate between improved agronomic practices and control. Whereas agronomic practices had larger contribution to total gain in yield than genetic improvement in this study, future assessment of agronomic and genetic gains in yield is warranted. Such assessment should focus more on rainfed rice systems, where agronomic gain was small, take into account genetic improvement rate over time and integrated agronomic practices rather than single intervention like nutrient management practice only, and be conducted in farmers’ fields.

## Highlights

• A meta-analysis was done to quantify genetic and agronomic gains in rice yield in SSA.
• Mean agronomic gain in yield of check variety was 0.8 t/ha.
• Mean genetic gain in yield was up to 0.9 t/ha.
• Genetic gain was 70% higher under improved practices than in control.
• Higher agronomic gain was associated with higher N application rate.

## 1 Introduction

Rice (*Oryza* spp.) is an important staple crop that plays a vital role in food security in sub-Saharan Africa (SSA), where its consumption has substantially increased over several decades and this trend is expected to continue Arouna et al., 2021; GRiSP, 2016; USDA FAS, 2019). Currently, the amount of rice imports in SSA is about 15.5 million metric tons and is expected to grow with increasing demand (Nigatu et al., 2017, USDA FAS, 2019). To meet present and future demand for rice in SSA, paddy yield, also called un-milled rice, which is much lower than the global average (2.2 t/ha vs. 4.6 t/ha, FAO, 2020), needs to be enhanced (Futakuchi et al., 2021, Saito et al., 2018, Saito et al., 2021, Tilman et al., 2011, Yuan et al., 2021).

Over more than 50 years, research for development (R4D) efforts made by a wide range of agricultural research organizations have concentrated on genetic improvement and agronomy for enhancing rice yield in SSA (Futakuchi et al., 2021, Saito et al., 2021, Tollens et al., 2013). An increase in yield through genetic improvement is referred to as “genetic gain in yield,” which is defined as yield increase through use of new variety and calculated by yield difference between new variety and variety popularly grown in the target site (Saito et al., 2021, Xu et al., 2017). It has been reported that newly released or newly developed breeding lines often show higher yield than old varieties when grown under same conditions in SSA (Saito et al., 2012). Based on recent field studies, Futakuchi et al. (2021) summarize genetic gain in yield in four target agroecosystems in SSA, and report average genetic gains of 0.6, 0.4, –0.4, and −0.5 t/ha in upland rice in West Africa, lowland rice in West Africa, irrigated lowland rice in the Sahel, and upland rice in the central highlands of Madagascar, respectively.

Meanwhile, Saito et al. (2021) defined agronomic gain in yield as the difference in yield between improved agronomic practices such as alternate nutrient, water, and weed managements, and/or their combinations and the control, which can be typical farmers’ practices, recommended practices, or practices having no input (e.g., no applied fertilizer). Several review papers have summarized impact of improved agronomic practices on agronomic gain in yield in SSA (Chivenge et al., 2021, Chivenge et al., 2022, Ibrahim et al., 2021, Saito et al., 2021). For instance, integrated crop management practices for irrigated rice systems showed yield gain from 1.6 to 3.1 t/ha (Saito et al., 2021), while improved nutrient management has resulted in agronomic gain in yield from 0.4 to 2.9 t/ha (Ibrahim et al., 2021). Site-specific nutrient management practices have been shown to increase yield gain on average by 0.5 t/ha (Chivenge et al., 2021). While the benefits of genetic improvement and improved agronomic practices for rice in SSA have been separately demonstrated in numerous studies, a comprehensive and systematic synthesis of impact of improved varieties and agronomic practices, and their interaction, on rice yield in SSA is lacking. Here we present the first quantitative synthesis of assessment of genetic and agronomic gains in yield and the contributions of genetic improvement and improved agronomic practice to yield gain and discuss major lessons learned. The findings of this study are expected to assist in directing future R&D efforts to satisfy the increasing rice demand in SSA.

## 2 Concepts of genetic and agronomic gains in yield

**Fig. 1:**
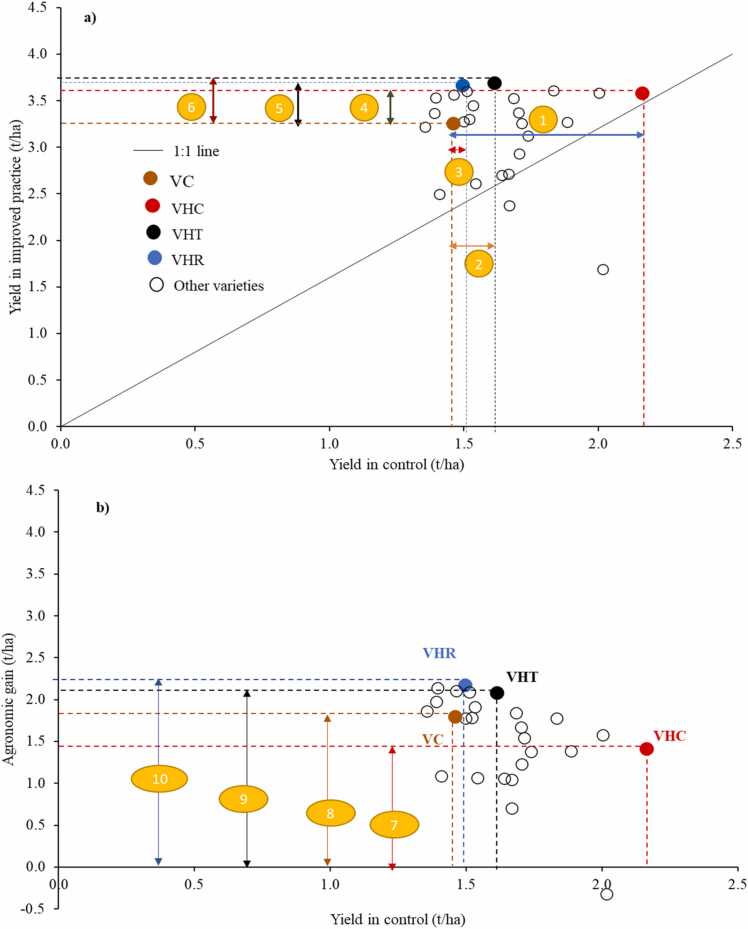
Illustration of selection of four types of varieties consisting of check variety [VC, see a), b)]; variety with highest yield in the control [VHC, see a), b)]; variety with highest yield under improved agronomic practices [VHT, see a)], and variety with largest yield difference between improved agronomic practices and control [VHR, see b)] using data on 26 varieties from Jones et al. (1997). For numbers (1−10) in ellipse with orange color, refer to Table 1.

This study focuses on field experiments consisting of variety and agronomic treatments for assessing genetic and agronomic gains in yield. We define genetic gain in yield as yield increase through use of new variety and calculated by yield difference between new variety and variety popularly grown in the target site, which is often used by trials conducted by both breeders and agronomists in the given sites and/or is popularly grown there (Saito et al., 2021, Xu et al., 2017). Difference in yield between improved agronomic practices such as alternative nutrient, water, and weed management practices, and/or their combinations and the control, which can be farmers' practice, practice with no external input (e.g., no fertilized condition), or recommended practice is defined as agronomic gain in yield (Saito et al., 2021). To compare between two gains in yield, we consider absolute/relative genetic gain in yield between high-yielding varieties and check which were tested in same trials. Here, we do not consider genetic gain per year or genetic improvement rate over time because our database included both released varieties and non-released, breeding pipelines. We also have some traditional varieties and ones introduced from Asia. As a result, calculating genetic improvement rate over time is not possible. In each trial, we measured total yield gain (i.e., difference between the control with check variety and improve agronomic practice with high-yielding variety). We purposefully identified four types of varieties in the study where many varieties were tested as illustrated in Fig. 1. The type of varieties identified are: (1) check variety (VC) which was generally indicated in each study, (2) variety with highest yield in the control (VHC), (3) variety with highest yield under improved agronomic practices (VHT), and (4) variety with largest yield difference between improved agronomic practices (referred to as "treatment") and control (VHR). With this approach, there is no negative genetic gain in yield of VHC in control and VHT in improved agronomic practices. We recognize that this approach could not have genetic gain over time and overestimated the genetic gain in yield which was typically accessed in multi-locational trials using same set of varieties as our hypothetical varieties were different across observations.

The agronomic practices included in this meta-analysis consisted of inorganic nitrogen (N), phosphorus (P), potassium (K), NPK, NPK + gypsum, and NPK + zinc fertilizers, organic amendment, crop establishment, water management, and weed management (Table 2). However, depending on the trial objectives, control treatment for agronomic practices, included typical farmers’ practices, recommended practices, or practices having no input (e.g., no applied fertilizer). As agronomic gain could be different, depending on type of control treatments (i.e., agronomic gain could be higher with no input than recommended practices), we considered three types of controls depending on trials. First, we considered control without external input or nutrient omission as C1. Examples included no fertilizer applied or nitrogen (N), phosphorus (P), and potassium (K) which were omitted from the NPK treatment (–N, –P, and –K plots). Second, control with recommended practices were considered as C2. Finally, farmer practice or low input level than recommended management practice (e.g., lower fertilizer input level) were considered as C3.

## 3 Material and methods

### 3.1 Selection of studies and database compilation

The literature search was conducted up to September 4, 2021, using Science Direct database, Google Scholar, ResearchGate, and AfricaRice publications database, using the search terms: “rice” OR “rice varieties” OR “rice genotypes” OR “varieties” AND “yield” AND “agronomic practices” AND “fertilizer” AND “nutrient” AND “nitrogen” AND “phosphorus” AND “potassium” AND “crop management” AND “water” AND “weed” AND “sub-Saharan Africa.” Studies included were peer-reviewed journal publications and authors’ own unpublished data that assessed impacts of variety, agronomic practice, and their interaction on rice yield in SSA. We excluded studies that had fewer than three varieties (e.g., Saito et al., 2010; Vandamme et al., 2018), or similar type of varieties without control or check variety (e.g., upland NERICA varieties only). Studies were rejected if the experimental method was not clearly described (e.g., experimental design was not defined, no variety by agronomic practice interaction is not properly assessed). If data from the same experiments were reported in multiple publications, the paper with the most complete dataset was used. Following these criteria, we extracted 208 observations from 40 studies covering 12 SSA countries. Relevant data collected from each study, include country, location, geographic coordinates; year of publication; year of experimental trial, production system (irrigated lowland, rainfed lowland, rainfed upland), agronomic practice, rate of fertilizer (N, P, and K) applied, yield, number of replications, and significance level of studied factors from analysis of variance, check varieties names. The check variety was mostly indicated in the publications. Otherwise, popular variety was identified by the authors. Detailed information on the compiled studies is provided in the Supplementary information (S1). Data on yield were extracted from table or figure in each study. When the data were presented in graphical formats, data were digitized using the GetData Graph digitizer V2.26.0.20 software. With the above approach, we compiled a database from 40 field studies conducted in 12 SSA countries, published between 1997 and 2021 (Supplementary information S1). A total of 208 paired observations were retrieved from these studies.

### 3.2 Calculations of genetic and agronomic gains in yield

In each observation, total yield gain and genetic and agronomic gains in yield of each variety type were calculated as indicated in Table 1.

**Table 1:** Total yield gain and genetic and agronomic gains in yield.

N°	Variable	Equation	Description
1	Genetic gain in yield of VHC in control	yield of VHC–yield of VC in control	Difference in yield between variety with highest yield in control and check variety in control
2	Genetic gain in yield of VHT in control	yield of VHT–yield of VC in control	Difference in yield between variety with highest yield in treatment and check variety in control
3	Genetic gain in yield of VHR in control	yield of VHR–yield of VC in control	Difference in yield between variety with highest responsiveness and check variety in control
4	Genetic gain in yield of VHC in treatment	yield of VHC–yield of VC in treatment	Difference in yield between variety with highest yield in control and check variety in treatment
5	Genetic gain in yield of VHR in treatment	yield of VHR–yield of VC in treatment	Difference in yield between variety with highest responsiveness and check variety in treatment
6	Genetic gain in yield of VHT in treatment	yield of VHT−yield of VC in treatment	Difference in yield between variety with highest yield in treatment and check variety in treatment
7	Agronomic gain in yield of VHC	yield of VHC in treatment−yield of VC in control	Difference in yield of variety with highest yield in control between treatment and control
8	Agronomic gain in yield of VC	yield of VC in treatment−yield of VHC in control	Difference in yield of check variety between treatment and control
9	Agronomic gain in yield of VHT	yield of VHT in treatment−yield of VHT in control	Difference in yield of variety with highest yield in treatment between treatment and control
10	Agronomic gain in yield of VHR	yield of VHR in treatment−yield of VHR in control	Difference in yield of variety with highest responsiveness between treatment and control
11	Total yield gain	yield of VHT in treatment−yield of VC in control	Difference in yield between variety with highest yield in improved agronomic practice (treatment) and check variety in control

check variety (VC), variety with highest yield in the control (VHC), variety with highest yield under improved agronomic practices (VHT), and variety with largest yield difference between improved agronomic practices and control (VHR).

### 3.3 Statistical analysis

The raw mean difference between treatment mean (*m*_*t*_) and control mean (*m*_*c*_) was calculated as effect size (*r*) using the *escalc* function in the *metafor* package in R statistical program version 4.0.2 (R Development Core Team, 2020). (Hedges, 1981). Since within-study variance measure was not reported in number of studies, we used replication approach to weight individual observations (Adams et al., 1997). Meta-analysis was performed using a random-effects model. Effect size and variance were included in the random-effects model using the *rma* function in *metafor* package. Random-effects model (with restricted maximum likelihood estimation) was used as we expected considerable heterogeneity between studies. The *weightfunct* in the *metafor* package was used to assess publication bias using the effect size and variance measures. The Likelihood Ratio Test revealed no evidence of publication bias. The effect size (*r*) was transformed to percent difference to express the results as follows:

% difference = (*r* x 100)/*m*_*c*_(12)

where *r* is the effect size while m_c_ is mean for the control.

To quantify source of variation in the data set – in other words, the proportion of variance explained by genetic and agronomic gains, and their interaction on total yield gain, we performed analysis of variance using mixed linear model. We considered two varieties (VHT and VC) and agronomic practices (improved treatment and control) as fixed factor, whereas observation was treated as a random factor.

We assessed the influence of inorganic N and NPK fertilizer management practices on agronomic gain using multiple linear regression with categorical explanatory variables (production system, season, variety type) and continuous independent variables (initial N fertilizer rate; N difference between two treatments). These treatments were selected as they had a higher number of observations than other agronomic practices. All analyses were conducted in the R statistical software version 4.0.2 (R Development Core Team, 2020).

## 4 Results

### 4.1 Description of dataset

Senegal accounted for the largest proportion (28 %) of studies, followed by Côte d′Ivoire (23 %) and Benin (13 %). Among the studies, 41 %, 34 %, and 25 % were from irrigated lowland, rainfed lowland, and rainfed upland rice, respectively, and 70 % were from wet season (Table 2). Seventy percent of the studies reported in this paper were conducted on research stations. Inorganic fertilizer management practices accounted for 80 % of the studies, of which 38 % were N management (Table 2). The other agronomic practices included weed management (9 %), crop establishment (5 %), organic amendment (2 %), and water management (4 %). The controls with no input (C1), recommended practices (C2), and farmer practice or low input (C3) accounted for 38 %, 48 %, and 14 %, respectively. VHT was the same as VHC in 35 % of observations, whereas VHR and VHT were the same in 51 % (Table 2). Based on data on ANOVA summary reported in the publications, there were significant effects of variety, agronomic practice, and their interaction on rice yield in 60 %, 65 %, and 60 % of the publications, respectively (Supplementary data S2).

**Table 2:** Number of observations disaggregated by production system, season, and agronomic practice.

	No. of data points	No. of observations having same variety for VC and VHC	No. of observations having same variety for VC and VHT	No. of observations having same variety for VC and VHR	No. of observations having same variety for VHC and VHT	No. of observations having same variety for VHR and VHT	No. of observations having same variety for VHC and VHR
**Overall**	208	32	44	38	73	107	29
**Production system**							
IL	111	19	23	20	36	55	13
RL	36	5	6	7	11	17	3
RU	61	8	14	11	26	35	13
**Season**							
Dry season	62	17	12	11	17	27	5
Wet season	146	15	32	27	56	80	24
**Agronomic practice**							
Crop establishment	10	2	1	0	7	6	5
Fertilizer (N)	80	2	17	19	30	49	16
Fertilizer (P)	37	5	4	5	11	15	1
Fertilizer (K)	4	2	3	0	1	0	0
Fertilizer (NPK)	31	1	4	7	11	15	5
Fertilizer (NPK + Gypsum)	7	5	1	1	1	4	0
Fertilizer (NPK + Zn)	8	1	3	0	2	2	0
Organic amendment	4	11	2	1	1	3	1
Water	9	3	4	0	2	5	0
Weed	19	0	5	5	7	8	1

VC, check variety; VHC, variety with highest yield in the control; VHT, variety with highest yield under improved agronomic practices; VHR, variety with largest yield difference between improved agronomic practices and control.

### 4.2 Total yield gains and genetic and agronomic gain in yield

**Fig. 2:**
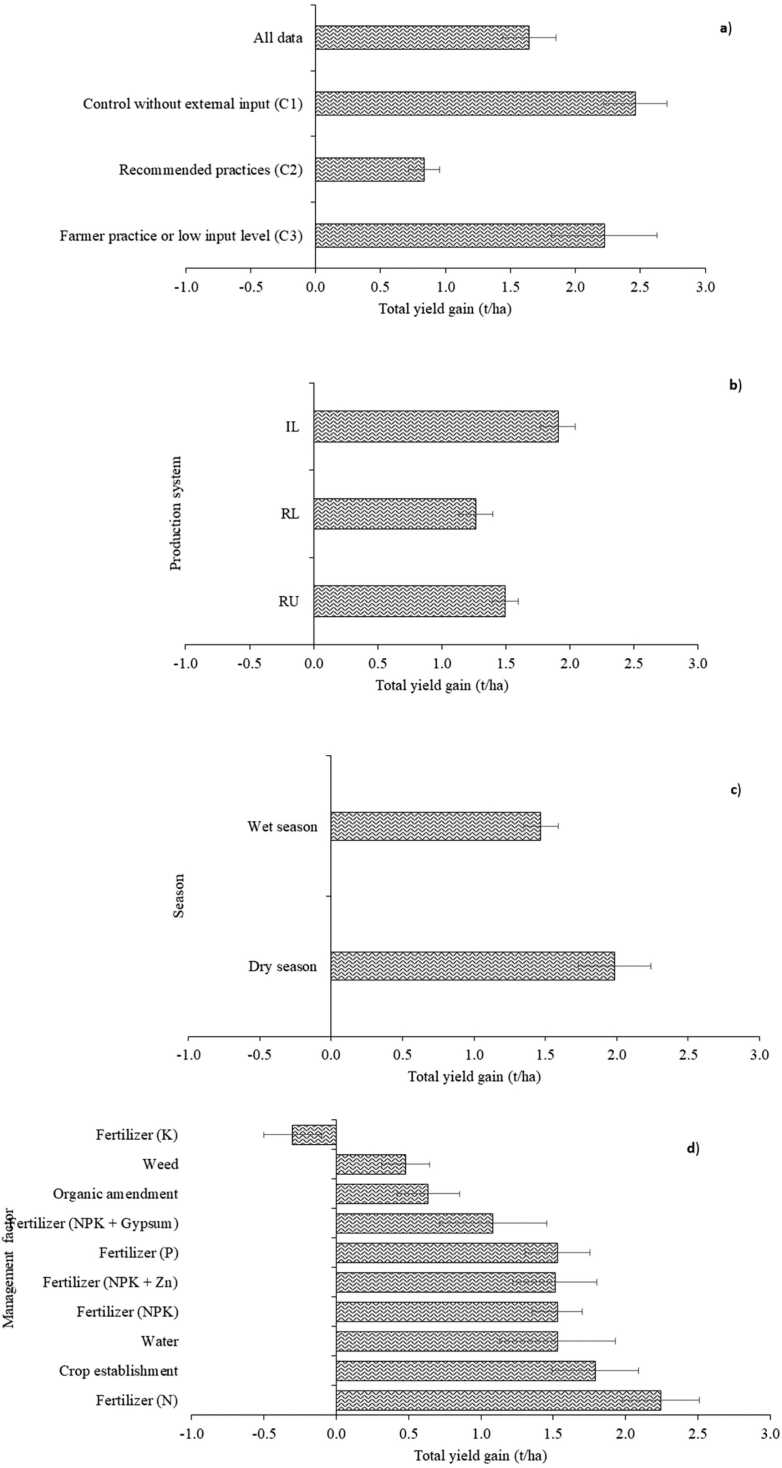
Total yield gain under a) all data point and control type, b) production systems, c) cropping seasons, d) management practices,.

On average, rice yields of VC in control were 5.0, 3.4, and 3.0 t/ha in irrigated lowland, rainfed lowland, and rainfed upland rice production systems, respectively (Table 2). Similarly, rice yields of VHT in improved agronomic practice were 6.8, 4.9, and 4.5 t/ha. Thus, the total yield gain (i.e., difference in yield between control with VC and improved agronomic practice with VHT) was 1.9, 1.3, and 1.5 t/ha, respectively, with average of 1.6 t/ha (Fig. 2). Total yield gain was higher in dry seasons than in wet seasons. K-fertilizer application, weed management, and organic amendment had lower total yield gain than other agronomic practices (N-fertilizer, crop establishment, water, NPK-fertilizer, fertilizer (NPK + Zn), P-fertilizer, fertilizer (NPK + gypsum). When data were disaggregated by type of control, total yield gain tended to be smaller with recommended practice (C2) than with no input (C1) and farmers’ practice (C3). When ANOVA was performed to quantify contribution of variety, agronomic practice and their interaction on yield, agronomic practice accounted for 75% of the total variation with variety and agronomic practice by variety responsible for 19 % and 6 %, respectively (Table 3).

**Table 3:** Yield in control and under improved practices.

	Yield in control (t/ha) (n = 208)				Yield in improved practices (t/ha) (n = 208)			
	VC	VHC	VHT	VHR	VC	VHC	VHT	VHR
**Overall**	4.1 ± 0.2	4.8 ± 0.2	4.4 ± 0.2	3.8 ± 0.2	4.9 ± 0.2	5.3 ± 0.2	5.8 ± 0.2	5.5 ± 0.2
**Production system**								
IL	5.0 ± 0.2	5.8 ± 0.2	5.3 ± 0.2	4.4 ± 0.2	6.0 ± 0.2	6.3 ± 0.2	6.8 ± 0.2	6.4 ± 0.1
RL	3.4 ± 0.3	4.0 ± 0.4	3.5 ± 0.4	2.9 ± 0.3	4.2 ± 0.4	4.5 ± 0.4	4.9 ± 0.4	4.5 ± 0.4
RU	3.0 ± 0.2	3.7 ± 0.3	3.3 ± 0.3	2.9 ± 0.3	3.5 ± 0.2	4.0 ± 0.3	4.5 ± 0.3	4.2 ± 0.3
**Season**								
Dry season	6.0 ± 0.4	6.5 ± 0.3	6.0 ± 0.3	5.4 ± 0.3	7.0 ± 0.3	7.3 ± 0.3	8.0 ± 0.3	7.7 ± 0.1
Wet season	3.4 ± 0.2	4.1 ± 0.2	3.7 ± 0.2	3.1 ± 0.2	4.0 ± 0.2	4.5 ± 0.2	4.8 ± 0.2	4.5 ± 0.2
**Agronomic practice**								
Crop establishment	5.6 ± 0.6	7.2 ± 1.0	7.0 ± 1.0	5.8 ± 1.1	6.0 ± 0.5	7.4 ± 1.0	7.7 ± 0.9	6.8 ± 0.9
Fertilizer (N)	4.9 ± 0.3	4.8 ± 0.3	4.4 ± 0.3	4.0 ± 0.3	5.3 ± 0.3	5.7 ± 0.3	6.1 ± 0.3	5.9 ± 0.3
Fertilizer (P)	3.9 ± 0.2	4.9 ± 0.3	4.2 ± 0.3	3.5 ± 0.2	4.5 ± 0.3	5.2 ± 0.3	5.7 ± 0.3	5.4 ± 0.3
Fertilizer (K)	6.9 ± 1.2	7.0 ± 1.2	6.8 ± 0.9	4.4 ± 0.7	6.0 ± 0.8	6.4 ± 0.5	6.3 ± 1.0	5.3 ± 0.8
Fertilizer (NPK)	4.1 ± 0.4	4.4 ± 0.4	4.2 ± 0.4	3.7 ± 0.4	5.2 ± 0.4	5.4 ± 0.5	5.7 ± 0.5	5.5 ± 0.5
Fertilizer (NPK + Gypsum)	4.9 ± 1.1	5.4 ± 0.8	5.3 ± 0.8	4.8 ± 1.1	5.4 ± 0.8	5.2 ± 0.6	5.7 ± 0.7	5.5 ± 0.8
Fertilizer (NPK + Zn)	3.8 ± 1.1	4.3 ± 1.0	4.1 ± 0.9	3.4 ± 0.8	4.5 ± 0.8	4.5 ± 0.8	5.0 ± 0.7	4.7 ± 0.7
Organic amendment	2.8 ± 0.3	3.0 ± 0.2	2.9 ± 0.2	3.0 ± 0.2	3.4 ± 0.4	3.4 ± 0.2	3.8 ± 0.3	3.8 ± 0.3
Water	5.1 ± 0.8	5.8 ± 1.0	4.9 ± 0.9	4.4 ± 1.0	6.1 ± 1.0	6.0 ± 1.0	6.6 ± 1.2	6.5 ± 1.2
Weed	3.2 ± 0.6	3.8 ± 0.7	3.4 ± 0.6	2.6 ± 0.5	3.1 ± 0.5	3.3 ± 0.6	3.7 ± 0.6	3.3 ± 0.2

VC, check variety; VHC, variety with highest yield in the control; VHT, variety with highest yield under improved agronomic practices (treatment); VHR, variety with largest yield difference between improved agronomic practices and control. Mean ± standard error; n, number of observations

**Fig. 3:**
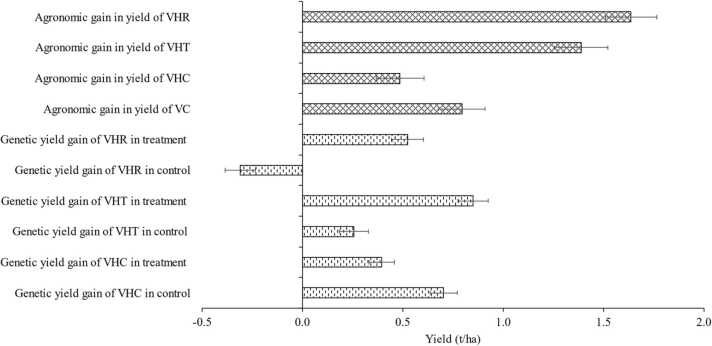
Agronomic and genetic yield gains. VC, check variety; VHC, variety with highest yield in the control; VHT, variety with highest yield under improved agronomic practices (treatment); VHR, variety with largest yield difference between improved agronomic practices and control.

Average yields of VC, VHC, VHT, and VHR were 4.1, 4.8, 4.4, and 3.8 t/ha in control, respectively, whereas they were 4.9, 5.3, 5.8, and 5.5 t/ha in improved agronomic practices (Table 2). Thus, genetic gains in yield of VHC, VHT, and VHR were 0.7, 0.3, and −0.3 t/ha in control, and 0.4, 0.9, and 0.5 t/ha in improved agronomic practices (Fig. 3). Agronomic gains in yield averaged 0.5, 0.8, 1.4, and 1.6 t/ha in VHC, VC, VHT, and VHR (Fig. 3).

### 4.3 Impact of agronomic practices on genetic gain in yield

**Fig. 4:**
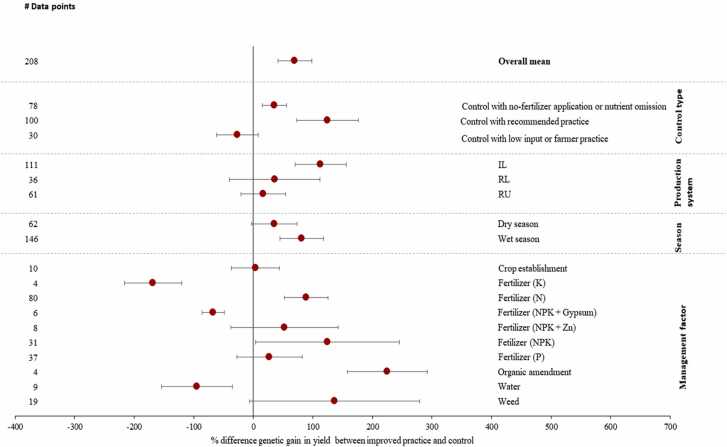
Genetic gain in yield of VTH in improved agronomic practice compared with genetic gain in yield of VTH in control. VHT, variety with highest yield under improved agronomic practices (treatment). Differences are expressed as mean percentage with 95 % confidence intervals represented by error bars. The number of observations is given as “# data points.

On average, the genetic gain in yield of VHT with improved agronomic practices was larger by 70 % than that in control (Fig. 4). Greater gain was observed in irrigated lowland rice (Fig. 3). Genetic gain in yield of VHT in improved practices with NPK fertilizer with gypsum application, K fertilizer application, and water management practices was smaller than that in control. However, these practices had relatively smaller samples (Fig. 4).

### 4.4 Agronomic gain vs. genetic gain

**Fig. 5:**
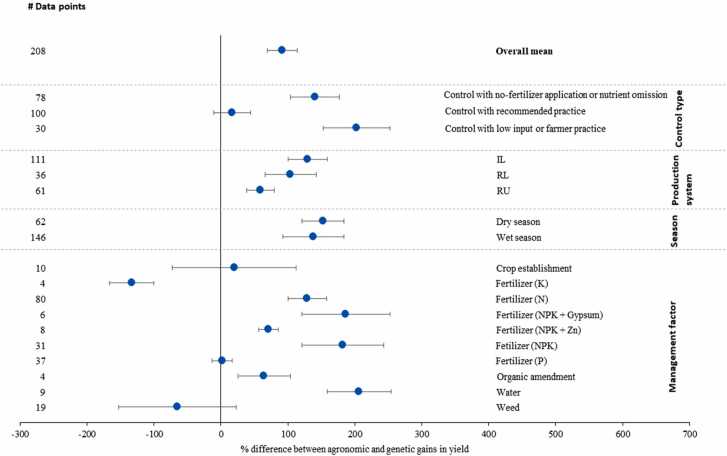
Agronomic gain in yield of VHT compared with genetic gain in yield of VHT in improved agronomic practice. VHT, variety with highest yield under improved agronomic practices (treatment). Differences are expressed as mean percentage with 95% confidence intervals represented by error bars. The number of observations is given as “# data points.”.

Here, we compared agronomic gain in yield of VHT and genetic gain in yield of VHT in improved agronomic practice, since genetic gain in yield of VHT tended to be larger than that in the control (see above sub-section). Agronomic gain was higher on average by 92 % than genetic gain (Fig. 5). Larger agronomic gain in yield was observed more in irrigated lowland rice than others. Both wet and dry seasons had higher agronomic gain. Greater genetic gain was observed when K fertilizer application and weed management practices were tested. Furthermore, agronomic gain was smaller when recommended practices (C2) were used as control than others. This is mainly due to the fact that agronomic gain in yield was smaller when improved practices were compared with recommended practices.

### 4.5 Impact of agronomic practice and variety on agronomic gain in yield

**Fig. 6:**
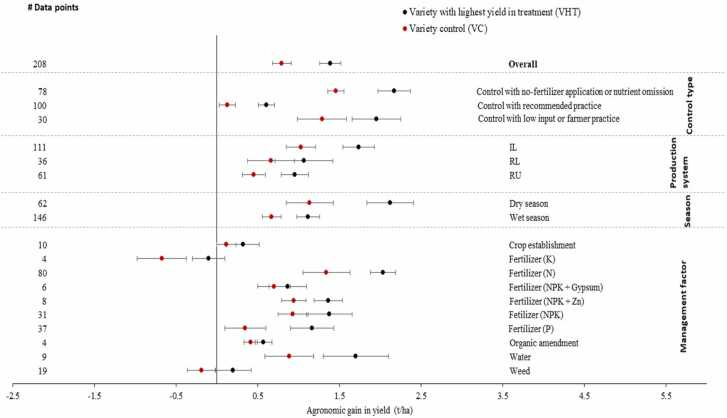
Agronomic gain in yield of VHT and VC in improved agronomic practice; VC, check variety; VHT, variety with highest yield under improved agronomic practices treatment.

The agronomic gain in yield of VC was on average 0.8 t/ha (Fig. 6). Average agronomic gains in yield of VC were 1.0, 0.7, and 0.5 t/ha in irrigated lowland, rainfed lowland, and rainfed upland, respectively, and higher in dry season than wet season (Fig. 6). Agronomic gain was negative with K fertilizer application and weed management practice. Agronomic gain in yield of VC in the control was smaller with recommended practice (C2) than with no-fertilizer input (C1) and farmers’ practice (C3). VHT consistently had higher agronomic gain in yield than VC regardless of production system, season, agronomic practices, and types of controls (Fig. 6).

### 4.6 Relationship between fertilizer application and agronomic gain

**Fig. 7:**
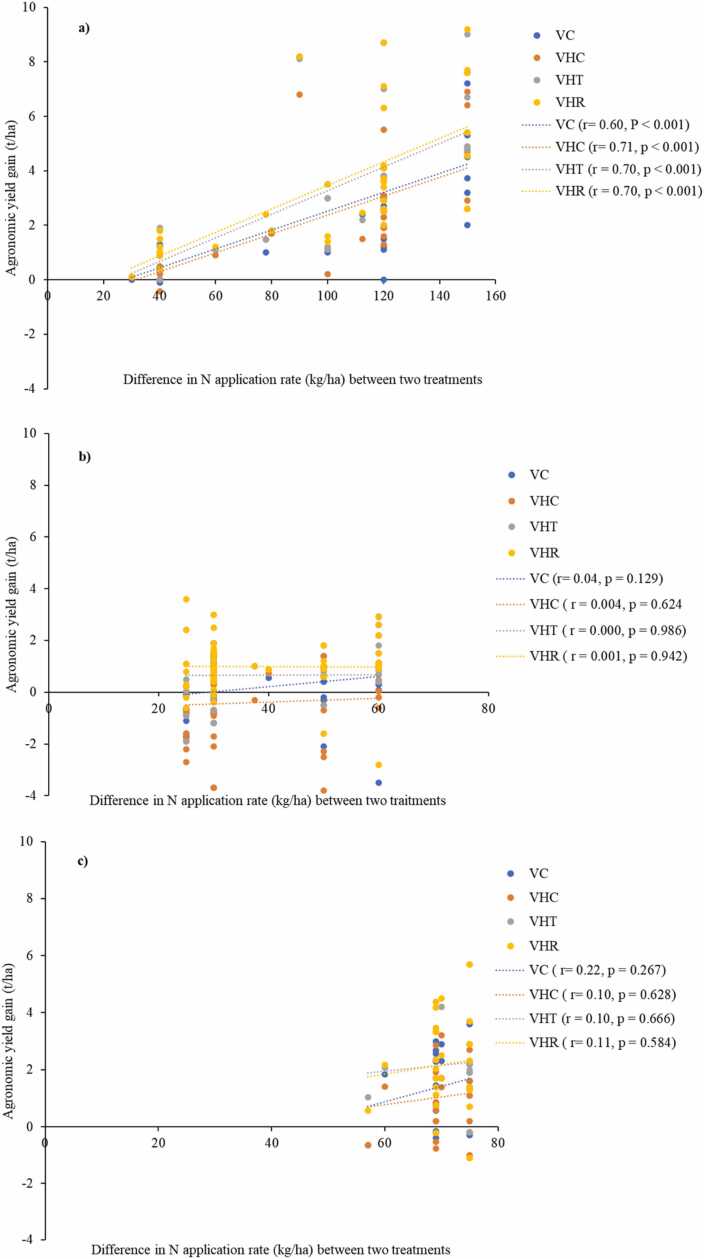
Relationship between agronomic gain in yield and difference in N application rate between control and improved agronomic practices (a) Control with no-fertilizer application or nutrient omission, (b) control with recommended practice, (c) Control with low input or farmer practice. VC, check variety; VHC, variety with highest yield in the control; VHT, variety with highest yield under improved agronomic practices (treatment); VHR, variety with largest yield difference between improved agronomic practices and control.

As inorganic fertilizer management practices accounted for more than 70 % of observations and these were dominated by inorganic N fertilizer management practice, we evaluated effects of N and other macronutrient (P, K) application rates on agronomic gain in yield. The difference in N application rate between no input as control (C1) and improved agronomic practice was positively correlated with agronomic gain in yield (Fig. 7a) but not between other controls and improved agronomic practices (Fig. 7b, c). Multiple regression analysis indicated that control type, N rate, and variety type were determinants for agronomic gain in yield. The agronomic gain in yield tended to be larger when: control was no fertilizer applied or N, P, K omitted from NPK fertilizer treatment; with increasing N rate; and with VHR and VHT (Table 4). Regression coefficient of N difference between two treatments was 0.019 t/kg, implying that agronomic fertilizer use efficiency was 19 kg/kg N. The coefficients of VHR and VHT indicated that replacing VC with these varieties would result in gains of 0.8 and 0.6 t/ha, respectively.

**Table 4:** Proportion of variance explained and F values for the effects of variety, agronomic practice, and variety x agronomic interaction on total yield gain.

Source of variation	Proportion of variance explained	F value
Variety	19.31	20.61***
Agronomic practice	75.13	80.19***
Variety x agronomic practice	5.55	5.93*

**Table 5:** Combined trials multiple regression analysis to assess the degree of influence of production system, management practice, N difference between two treatments, control type, and variety type on agronomic gain in yield using data from inorganic fertilizer management practices.

Dependent variable	Independent variable	Estimate	t value	Adjusted R-squared	Residual standard error	F-value
Agronomic gain in yield	Production system: Rainfed lowland^§^	–0.690	–2.167*	0.45	1.53	42.14***
	Production system: Rainfed upland^§^	–0.950	–3.581***			
	Management practice: Fertilizer (NPK)^§§^	0.210	1.144***			
	Control type (recommended rate)^§§§^	–1.674	–6.933***			
	Control type (farmers ‘practice)^§§§^	-0.697	-3.247***			
	N difference between two treatments	0.019	6.897***			
	Variety: VHC^‡^	–0.331	–1.641			
	Variety: VHR^‡^	0.641	3.172**			
	Variety: VHT^‡^	0.814	4.028***			

^§, §§, §§§, ǂ,^ Irrigated lowland, fertilizer (N), control without fertilizer application, and VC are references for production system, management practice, trial type, and variety type, respectively.

***, ** denote statistical significance at the 0.001, 0.01 levels, respectively.

VC, check variety; VHC, variety with highest yield in the control; VHT, variety with highest yield under improved agronomic practices (treatment); VHR, variety with largest yield difference between improved agronomic practices and control.

## 5 Discussion

This study is the first meta-analysis quantifying both genetic and agronomic gains in rice yield in sub-Saharan Africa (SSA). Our observations are dominated by irrigated lowland rice production systems. The distribution of studies (41 %, 34 %, and 25 % from irrigated lowland, rainfed lowland, and rainfed upland rice) across production systems is not entirely proportional to the distribution of estimated area under rice over these three systems (26 %, 38 %, 32 %) (Diagne et al., 2013). This is partly because AfricaRice Sahel regional research stations in Senegal have had research focus on irrigated rice since 1976, and a lot of efforts has been made to develop improved agronomic practices and varieties (Futakuchi et al., 2021, Ibrahim et al., 2021). Furthermore, higher share of studies conducted in Côte d′Ivoire and Benin in this paper is also due to the fact of existence of AfricaRice research stations (for Benin, 2005–2017). In agronomic practices, inorganic fertilizer management practices accounted for 78% of the studies, of which 48 % were nitrogen (N) management. An increasing emphasis on nutrient management in rice research in SSA could be attributed to the fact that soil nutrient deficiencies are generally considered a major cause of low yield and large yield gaps of rice in SSA (Bado et al., 2018, Saito et al., 2019). Although previous studies clearly showed large yield gain with integrated agronomic practices consisting of more than two component technologies including nutrient, water, and weed management practices (Ibrahim et al., 2021, Senthilkumar et al., 2018), we found limited studies on integrated agronomic practices in our database (e.g., Krupnik et al., 2012; Oikeh et al., 2009). Furthermore, most studies were conducted on research stations. Due to the wide range of constraints observed in farmers' fields that cannot be simply addressed with one single component technology (Dossou-Yovo et al., 2020, Niang et al., 2017), there is a need to evaluate the combined impact of integrated agronomic practices and improved varieties in farmers' fields to assess agronomic and genetic gains, as well as their interaction in the future studies.

Our finding of larger contribution of agronomic practice to variation in yield than genetic improvement is compatible with previous studies on rice in Japan and on maize in US (Horie et al., 2005, Rizzo et al., 2022), which showed limited contribution of genetic improvement in yield potential to yield increase in farmers’ fields. The variation in total yield gain was associated with production system, management factor, and season. It is well known that irrigated lowland rice especially in dry season tended to produce higher yield with its higher potential yield and better response to external inputs such as nutrient and water in the tropical environments (Kropff et al., 1993). Some of agronomic practices, e.g., potassium fertilizer, had lower total yield gains than others. Lower yield response to potassium fertilizer is supported by results from on-farm nutrient omission trials for rice in SSA (Saito et al., 2019), showing that rice yields without N, P, and K were 68 %, 84 %, and 89 % of yields in the NPK treatment, respectively.

The ranges in genetic gain in yield observed in this study (from –0.3–0.7 t/ha in control for means of VHC, VYH, and VHR, compared with 0.4–0.9 t/ha under improved agronomic practices) are compatible with genetic gain in rice yield observed in recent variety evaluation trials in SSA (Futakuchi et al., 2021, Graham‐Acquaah et al., 2018, Rodenburg et al., 2009, Saito, 2016, Saito et al., 2014, Saito et al., 2018). VHT was identical to VHC in 35 % of observations, and VHR and VHT were the same in 51 % and the genetic gain in yield of VHT was higher in improved agronomic practices than in control (0.9 vs. 0.3 t/ha). These indicate that it is possible to develop varieties adapted to different agronomic practices and high-yielding varieties tend to be responsive to improved agronomic practices. This finding indicates that breeders could reduce the cost for developing different varieties adapted to specific agronomic practices and producing seeds for their dissemination. Our results agree with previous studies in Asia showing that improved rice varieties, combined with inputs of fertilizer and/or irrigation, have contributed to great yield increases in lowland and upland rice production systems (Evans, 1996, Saito et al., 2007). The greater genetic gain in irrigated lowland rice than in rainfed rice production systems in this study agreed with a recent study in India (Kumar et al., 2021), but was in contrast with a recent review paper that showed that irrigated lowland hybrid rice varieties had lower genetic gain than inbred check varieties in the Sahel of West Africa (Futakuchi et al., 2021).

Mean agronomic gains in yield ranged from 0.5 to 1.6 t/ha across variety types in this study. They were not markedly different from agronomic gain in yield observed with improved agronomic practices in the Sahel (Ibrahim et al., 2021) and across SSA countries (Saito et al., 2021). Higher agronomic gain in irrigated rice than rainfed lowland rice in this study is in line with previous studies in Asia and Africa (Matsunami et al., 2009; Vandamme et al., 2016). VHT consistently had a higher agronomic gain in yield than VC regardless of production system, season, agronomic practices, and types of controls. This suggests that using improved varieties yields more and generates higher marginal profits for farmers than using old, improved rice varieties (VC) released several decades ago (Futakuchi et al., 2021).

Agronomic gain in yield of VHT was higher than genetic gain in improved agronomic practices in 54 % of observations. This indicates that better gain in yield could be further achieved through combining improved varieties with improved agronomic practices. Higher agronomic gain in yield was also associated with larger difference in N application rate between improved agronomic practices and control. This indicates that appropriate N fertilizer application is required to improve rice yield in SSA. The present finding is consistent with the results of previous studies that showed that N is the major limiting macronutrient in rice production in SSA and that increasing N application leads to rice yield increment in all three major rice production systems (Asai et al., 2021, Saito et al., 2019; Tsujimoto et al., 2019). Furthermore, Yuan et al. (2021) identified several rice cropping systems exhibiting negative N balance in SSA, suggesting soil N mining over time. These systems would clearly benefit from a greater N input (Yuan et al., 2021). Agronomic yield gain is generally limited by both environmental (e.g., climatic variability) and socioeconomic (e.g. fertilizer cost) factors that influence farmers’ decision making for investment (Stuart et al., 2016).

## 6 Conclusion

This study demonstrates agronomic and genetic gains observed for rice in field trials in sub-Saharan Africa and presents the scope for improvement of rice yield through improved agronomic practices and genetic improvement. Whereas improved agronomic practices had larger contribution to total gain in yield than genetic improvement in this study, future assessment of agronomic and genetic gains in yield is warranted. We suggest that future research should focus more on rainfed rice systems, where agronomic gain was small, consider genetic improvement rate over time and integrated agronomic practices rather than single intervention like nutrient management practice only, and be conducted in farmers’ fields.

## CRediT authorship contribution statement

**Ali Ibrahim**: Papers search, conceptualization, methodology, data analysis, writing original draft preparation; **Saito Kazuki**: Papers validation, Investigation, writing review and editing, supervision, funding acquisition. All authors read and approved the final manuscript.

## Declaration of Competing Interest

The authors declare that they have no known competing financial interests or personal relationships that could have appeared to influence the work reported in this paper.

## Data Availability

Data will be made available on request.
