# Supplementary material for: Assessing genetic and agronomic gains in rice yield in sub-Saharan Africa: A meta-analysis
Source: Field Crops Res. 2022 Oct 15;287:108652. doi: 10.1016/j.fcr.2022.108652 (PMC9489921; doi:10.1016/j.fcr.2022.108652)
Supplement: Supplementary file 2 — Supplementary material [file mmc2.docx]

Supplementary Information (S2)


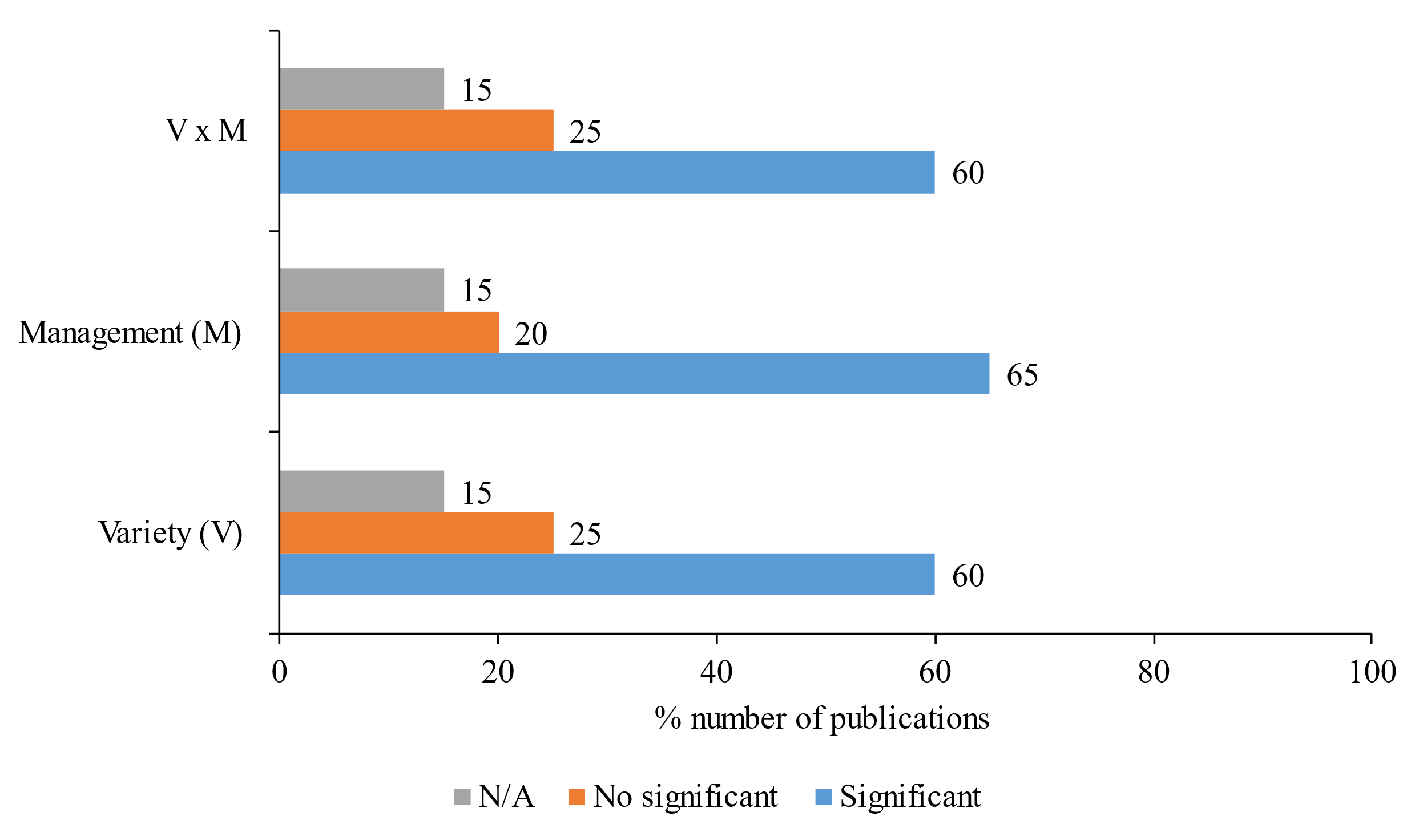


Fig. 1. Number of publications reported significant effect of variety, management and variety by management interaction.
